# Surface Tamm States of 2–5 nm Nanodiamond via Raman Spectroscopy

**DOI:** 10.3390/nano13040696

**Published:** 2023-02-10

**Authors:** Mikhail Popov, Fedor Khorobrykh, Sergei Klimin, Valentin Churkin, Danila Ovsyannikov, Alexander Kvashnin

**Affiliations:** 1 Technological Institute for Superhard and Novel Carbon Materials, 7a Tsentralnaya, 108840 Troitsk, Moscow, Russia; 2 Phystech School of Electronics, Photonics and Molecular Physics, Moscow Institute of Physics and Technology Institutskiy per. 9, 141700 Dolgoprudny, Moscow, Russia; 3 Scientific and Technological Center of Unique Instrumentation, Russian Academy of Sciences, Butlerova Str. 15, 117342 Moscow, Russia; 4 Institute of Spectroscopy RAS, Fizicheskaya Str. 5, 108840 Troitsk, Moscow, Russia; 5 Skolkovo Institute of Science and Technology, Bolshoy Boulevard 30, Bld. 1, 121025 Moscow, Russia

**Keywords:** resonance Raman scattering, nanodiamond, phonon density of states, lattice dynamic

## Abstract

We observed resonance effects in the Raman scattering of nanodiamonds with an average size of 2–5 nm excited at a wavelength of 1064 nm (1.16 eV). The resonant Raman spectrum of the 2–5 nm nanodiamonds consists of bands at wavelengths of 1325 and 1600 cm^−1^, a band at 1100–1250 cm^−1^, and a plateau in the range from 1420 to 1630 cm^−1^. When excited away from the resonance (at a wavelength of 405 nm, 3.1 eV), the Raman spectrum consists of only three bands at 1325, 1500, and 1600 cm^−1^. It is important to note that the additional lines (1500 and 1600 cm^−1^) belong to the sp^3^-hybridized carbon bonds. The phonon density of states for the nanodiamonds (~1 nm) was calculated using moment tensor potentials (MTP), a class of machine-learning interatomic potentials. The presence of these modes in agreement with the lattice dynamics indicates the existence of bonds with force constants higher than in single-crystal diamonds. The observed resonant phenomena of the Raman scattering and the increase in the bulk modulus are explained by the presence of Tamm states with an energy of electronic transitions of approximately 1 eV, previously observed on the surface of single-crystal diamonds.

## 1. Introduction

The bulk modulus of a 2–5 nm nanodiamond is 607 GPa, which exceeds the value for a single-crystal diamond (443 GPa) [1,2]. Since the mechanical properties of the materials formed by covalently bonded carbon atoms are determined by their elastic moduli [3], the study of the effect of an increase in the elastic moduli with a decrease in the size of the nanocluster will explain the possible increase in the strength of nanopolycrystalline materials [4,5].

In accordance with the lattice dynamics, an increase in the bulk modulus of a nanodiamond should be accompanied by the appearance of bonds with force constants higher than that of a diamond and, accordingly, the appearance of Raman modes with frequencies higher than the single-crystal diamond frequency of 1333 cm^−1^. The simulations of diamond nanoparticles suggest that the observed high value of the bulk modulus is originated from shortened sp^3^ bonds in the near-surface region [2]. The Raman spectrum of a 2–5 nm nanodiamond consists of three bands at the frequencies of 1325, 1600, and 1500 cm^−1^ (laser excitation at 458 nm). The band at 1500 cm^−1^ shifts to 1630 cm^−1^ when excited by a laser beam with a wavelength of 257 nm [1]. It is important to note that the additional lines (1600 and 1500 cm^−1^) are observed for a 2–5 nm nanodiamond that are not contaminated with impurities as discussed in [1,2]. To prevent contamination of the surface, the nanodiamond must be dispersed in NaCl after annealing in a vacuum. The saturation of the surface of the diamond nanoparticles with hydrogen, for example, leads to the disappearance of these additional lines [6].

One of the typical features of nanodiamonds is the presence of a band at approximately 1600 cm^−1^. However, this band is mostly absent in Ref. [7] (with the exception of 1.3 and 1.4 nm nanodiamonds), which is probably due to the synthesis conditions of nanodiamonds of certain sizes, as a result of which diamonds are chemically bound to hydrogen. In the well-purified nanodiamonds studied at Ref. [8], the effect described in [1,2] was discovered. It was found that the relative intensities of the bands in the regions of 1600 cm^−1^ and 1325 cm^−1^ do not depend on the excitation wavelengths of 244–514 nm. In addition, the profile of the diamond line and the broad line at approximately 1250 cm^−1^ is not affected by the wavelength change. In Ref. [8], it is concluded that the band in the 1600 cm^−1^ region is caused by sp^2^ bonds and belongs to the so-called “G” line. It is noted that the origin of the “G” line of nanodiamonds is still unknown. One of the main arguments, based on which in Ref. [8] the 1600 cm^−1^ band was attributed to sp^2^ bonds, was that one cannot be attributed to any pure sp^3^ form of carbon, being far away from the cut-off frequency of the phonon density of states (PhDOS) of a diamond.

As shown in Refs. [1,2], the additional lines at 1600 and 1500 cm^−1^ refer exclusively to the sp^3^ bonds. This conclusion was reached based on the fact that the relative intensities of the Raman bands at 1325, 1500, and 1600 cm^−1^ do not depend on the excitation wavelength in the 257–532 nm range. Indeed, a scattering cross-section of sp^2^-bonded carbon exceeds the one of sp^3^-bonded carbon by a factor of 50–200 at a laser excitation in the visible range, and the cross-sections are mutually equal at the 257 nm laser excitation [9]. In our case, the absence of the resonance effect associated with the presence of sp^2^ bonds indicates the absence of sp^2^-bonded carbon. According to the data of both parallel electron energy loss spectroscopy and nuclear magnetic resonance spectroscopy [10,11], there is no sp^2^-bonded carbon in pure nanodiamonds.

The occurrence of states localized on the crystal surface or the boundary of two materials is generally associated with the braking of translational symmetry. The possibility of the existence of such states, in which electrons can be localized on the surface moving only along it, was theoretically predicted by I.E. Tamm in 1932 [12]. These electronic surface states are known as Tamm states.

For the single-crystal diamond, the surface states have been studied in [13,14,15]. An excitation from occupied surface states into unoccupied surface states was studied using electron energy loss spectroscopy (EELS) with the valence band excitation. The surface band gap was measured to be approximately 1 eV [13]. For a comparison, the gap for a bulk diamond is 5.5 eV [15]. A study performed by combining ultraviolet photoelectron spectroscopy (UPS) with EELS gavea surface band gap of 2 eV [14]. Later [15], the angle-resolved photoelectron spectroscopy study showed a gap of approximately 0.5 eV in the surface electronic structure. It is essential that the surface states of the diamond are observed only for clean surfaces. Saturation of the surface with hydrogen leads to the disappearance of the effects associated with the appearance of the surface band gap [13,14,15].

Resonant effects in Raman scattering are expected when excited with photon energies close to Tamm states (or other critical points of the interband transitions, e.g., Van Hove singularities) [16]. In this study, we present an observation of such resonances for 2–5 nm nanodiamonds in the Raman spectrum excited at a wavelength of 1064 nm (1.16 eV).

## 2. Materials and Methods

### 2.1. Experimental Details

Purified detonation 2–5 nm diamonds produced by the SANTA company (Brest, Republic of Belarus) were used for the study. The problem of an additional purification was discussed in detail in our previous studies. To prevent contamination and a deposition of water on the surface, the nanodiamonds, after the additional purification, were encapsulated in NaCl in the same manner as described in Refs. [1,2]. Briefly, the cleaning algorithm is as follows. After the manufacturer cleaned the nanodiamonds, we annealed them in a vacuum and then processed them in a planetary mill with a mixture of 25 wt. % of NaCl. The treatment mode was selected so as to replace the remnants of contamination on the surface of the nanodiamonds by NaCl (while the remnants of contamination diffused into the NaCl). After processing, the powder was pressed into a tablet in an inert atmosphere. As noted in [1,2], C-O bonds and O atoms were not observed according to the XPS data after the treatment. A Fritsch planetary mill with ceramic silicon nitride bowls and balls of 10 mm in diameter was used. The treatment of 20 min in the planetary mill provided homogeneous nanocomposites without contamination by the material of the ceramic balls.

The Raman spectra were recorded with a Renishaw inVia Raman microscope (excitation wavelength 405 nm), TRIAX 552 (Jobin Yvon Inc., Edison, NJ, USA) spectrometer equipped with a CCD Spec-10, 2KBUV Princeton Instruments 2048 × 512 detector and razor edge filters (excitation wavelength 257 and 458 nm), and Raman–Fourier spectrometer (RFS) 100/s (Bruker company, Germany), excitation wavelength 1064 nm. The spectra were acquired at a laser radiation power density of 10^8^ W/m^2^. Shifts of the line at 1325 cm^−1^ into the low-frequency region by more than 1 cm^−1^ due to the sample heating were observed at the laser radiation power density of more than 3 × 10^8^ W/m^2^.

We used a diamond anvil cell (DAC) for a high-pressure study. The nanodiamonds with the size of 2–5 nm encapsulated in NaCl were loaded into a prepared hole in a tungsten gasket. We used diamond anvils constructed of synthetic diamond with a high content (~60%) of ^13^C isotope. The use of these anvils made it possible to observe the 1325 cm^−1^ nanodiamond Raman band under pressure.

The pressure was measured from the stress-induced shifts of the Raman spectra from the diamond anvil [17]. Since we used diamond anvils constructed of synthetic diamond with a high content of ^13^C isotope, we performed an additional calibration of the diamond pressure scale described in Ref. [1].

### 2.2. Computational Details

Geometry optimization of the nanodiamond cluster with an average diameter of 0.9 nm (92 carbon atoms) was performed in the framework of the density functional theory [18,19] by using the VASP package [20,21,22] within the generalized gradient approximation (the Perdew–Burke–Ernzerhof functional) [23] and the projector augmented wave method [24,25]. The plane-wave energy cutoff was set to 400 eV. For the nanodiamond cluster, the Γ-point calculation was used. The distance between the periodically located cluster was set to be no less than 20 Å to avoid the artificial influence of the layers on each other. Atomic structure minimization was carried out until the change in total energy was less than 10^−4^ eV.

The calculation of the phonon densities of states by using the density functional perturbation theory or finite displacement method is resource-consuming for low-symmetry structures and even more so for non-periodic systems. Here, the resource-consuming DFT computations were replaced by calculations based on passively trained machine-learning interatomic potentials, namely the moment tensor potentials (MTPs) [26] that were trained by using the mlip package [27], showing an exceptionally high level of accuracy [28,29,30,31]. MTPs were trained over short ab initio molecular dynamics (AIMD) trajectories. We prepared the training sets by conducting AIMD simulations based on the density functional theory (DFT) [18,19] using similar parameters as those for geometry relaxation.

Two configuration sets were generated by using AIMD simulation at a constant temperature of 50 K and with a temperature reduction from 1000 to 200 K (2000 timesteps each). The time step for simulation was chosen to be equal to 1fs. More details about the training procedure required for the calculation of the MTP forces can be found in Ref. [27].

Force constants calculated by MTP (mlip_phonopy interface [32]) were used in the PHONOPY [33] package to evaluate the phonon dispersion curves and phonon densities of states.

## 3. Results and Discussion

**Figure 1:**
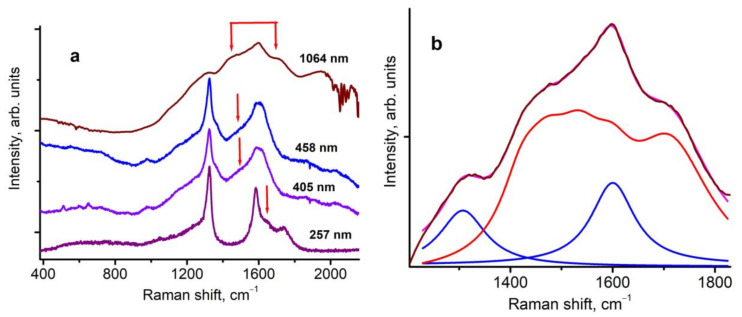
Raman spectra of the studied 2–5 nm nanodiamonds encapsulated in NaCl. (**a**) Raman spectra measured with four different excited wavelengths in the region of 257–1064 nm; red arrows mark the dispersive band position. (**b**) Lorentz multi-peak fits of Raman spectrum excited with 1064 nm.

The Raman spectra of the 2–5 nm nanodiamonds encapsulated in NaCl excited at wavelengths of 257, 405, 458, and 1064 nm are presented in Figure 1a. The spectra are marked by the corresponding excitation wavelengths. As we discussed earlier [1,2], the Raman spectra of a nanodiamond consists of three bands located at 1325, 1600, and 1500 cm^−1^ (with laser excitation of 458 nm). The band at 1500 cm^−1^ shifts to 1630 cm^−1^ when excited by a laser with a wavelength of 257 nm [1]. Also, a wide band from 1100 to 1250 cm^−1^ is observed in all the measured spectra.Usually, this band is referred to as disordered sp^3^ carbon [34]. The narrow lines observed at 1064 nm excitation (upper spectrum in Figure 1a) at a frequency slightly higher than 1960 cm^−1^ are due to the absorption of residual water vapor in the atmosphere (a band with the rotational structure centered at 1.38 μm).

The observed dispersion of the band between 1500 and ~1630 cm^−1^ is typical for various sp^3^ carbon clusters (for example, 3D C_60_, ultrahard fullerite, or nanodiamond) [1,2,35,36,37]. For diamond-like carbon, the peak dispersion linearly depends on the exciting wavelength in the range of 200–800 nm [38]. The resonant Raman spectra of tetrahedral amorphous carbon was calculated, and a model of resonant Raman spectra of carbon films was presented [38]. According to the model, the dispersed band is attributed to the G peak, which arises from chains of sp^2^-bonded atoms. Significantly, the model contains 28% of the sp^2^-bonded carbon atoms.

In practice, there is no sp^2^-bonded carbon in pure 2–5 nm nanodiamonds, according to both parallel electron energy loss spectroscopy and nuclear magnetic resonance spectroscopy data (the sensitivity of the methods is better than 1%) [10,11].No such chains were observed in the sp^3^ carbon clusters described in Refs. [1,2,35,36,37], although in all these works the effect of dispersion was observed, including 3D C_60_ with 92% of the sp^3^ bonds [35]. Moreover, the effect of the dispersion at a pressure up to 75 GPa was described in Ref. [35], when the presence of sp^2^ bonds in the sample at a pressure above 40 GPa is impossible [39]. Thus, in the case of sp^3^-bonded 2–5 nm nanodiamonds, the nature of the dispersion is not clear.

When excited with a wavelength of 1064 nm, the resonant Raman spectrum is observed (Figure 1a,b). Let us look at the Raman spectrum of 2–5 nm nanodiamonds when excited at a wavelength of 1064 nm in more detail. Lorentz multi-peak fits of the Raman spectrum excited at 1064 nm are plotted in Figure 1b. The bands at approximately 1325 and 1600 cm^−1^ are distinctly present in the spectra. A plateau (marked by the red color) that is composedof multi-bands in the range from 1423 to 1713 cm^−1^ is observed. The appearance of this plateau is a new feature of the Raman spectrum of 2–5 nm nanodiamonds, which manifests itself when excited at 1064 nm. The plateau covers all possible positions of 1500 to ~1630 cm^−1^ of the dispersed line, which it occupies when excited at wavelengths of 458–257 nm.

The relative intensities of the Raman lines also changed significantly when the wavelength of the exciting radiation changed from 458–257 nm to 1064 nm. In particular, the relative intensities of the bands at approximately 1325 and 1600 cm^−1^ and the plateau at the wavelength range 1423–1713 cm^−1^ as well as the band at 1100–1250 cm^−1^ are changed. When excited at 458 to 257 nm, the intensity of the band at approximately 1325 cm^−1^ is approximately 2-times higher than the intensities of the bands at 1100–1250 cm^−1^, the band at 1600 cm^−1^, and the bands at 1500–1630 cm^−1^. However, when excited at 1064 nm, the intensity ratio changes to the opposite (see Figure 1a). The intensity of the plateau and the band at 1600 cm^−1^ exceeds the intensity of the bands at 1325 cm^−1^ by a factor of 2 (see Figure 1b). Thus, with the resonant excitation, the relative intensities of the bands at 1100–1250 cm^−1^, 1600, and 1500–1630 cm^−1^ increased 4-fold, and additional lines appeared, which expanded the previously observed dispersion interval of 1500–1630 cm^−1^ to 1423–1713 cm^−1^. Thus, the full spectrum of the Raman-active excitations of the nanodiamonds under consideration consist of bands at 1100–1250 cm^−1^ and the plateau in the range from 1423 to 1713 cm^−1^.

Let us take a closer look at the 4-fold increase in the intensity of the Raman spectrum of bands in the range from 1423 to 1713 cm^−1^. Such an increase in the intensity of the spectrum with the change in the exciting wavelength has been described in detail within the framework of the concept of resonant Raman scattering [16]. In particular, resonance amplification of the spectrum is observed when the energy of the exciting radiation is near the absorption edge. In our case, the resonance is observed when excited at a wavelength of 1064 nm (1.16 eV). In terms of energy, this corresponds to the surface Tamm states with a band gap of approximately 1 eV [13,14,15], as we discussed above in the Introduction section.

The nature of the dispersion effect when only part of the plateau 1423–1713 cm^−1^ is excited, depending on the wavelength of the exciting radiation, is not yet clear. It should be noted that, when excited at 257 nm, the Raman line at 1630 cm^−1^ is observed, which in fact can be considered as close in frequency to the edge of the plateau. The higher frequency modes at approximately 1700 cm^−1^ are probably related to the second order. To clarify this issue, we undertook the calculation of the density of the phonon states of the nanodiamonds.

**Figure 2:**
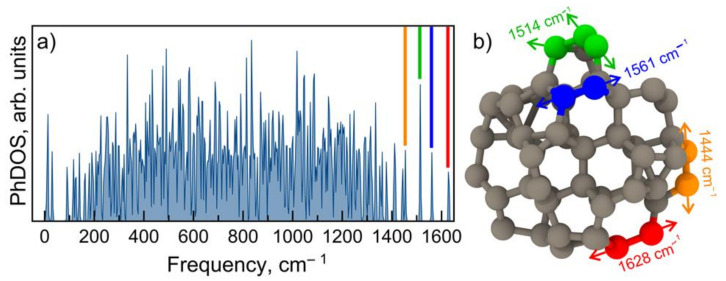
(**a**) Phonon density of states of considered nanodiamonds. (**b**) Visualization of atomic vibrations corresponding to high-frequency phonon modes. Colored bands in (**a**) display phonon modes, which are visualized in (**b**). Arrows denote the direction of atom oscillations.

Using a trained MTP for nanodiamonds, we calculated the phonon density of states (PhDOS) as shown in Figure 2a. It is clearly seen that the acoustic phonon modes are separated from the optical by a gap of approximately 70 cm^−1^.

The structure of the 0.9 nm cluster contains 92 carbon atoms, while the 2–5 nm cluster contains from 172 to 1370 atoms. The large number of atoms is a problem from a technical point of view to perform the relaxation of such big structures in DFT because only after DFT relaxation can the MTP potential be used to perform the calculations of the phonon DOS. However, we are interested in the high-frequency vibrations caused by the surface atoms that bounded to three neighboring atoms. For larger clusters, we will observe a larger number of the same high-frequency vibration modes. So, in principle, nothing will change with a change in the size;only the intensities of the high-frequency modes will decrease, while the frequencies will be approximately the same.

One can note the presence of high-frequency modes at ~1450–1650 cm^−1^. These modes logically should correspond to C-C bonds with higher strength (shorter bonds). Such atoms are located on the surface of the cluster. To prove this assumption, we have visualized these phonon modes as shown in Figure 2b. The color of the arrows corresponds to the phonon modes highlighted by the colored bands in Figure 2a. One can see that the mode with the highest frequency of 1628 cm^−1^ (red band in Figure 2a) is associated with the motion of the surface atoms, as was supposed. Other high-frequency vibrations also correspond to the motions of the surface atoms (see Figure 2b).

**Figure 3:**
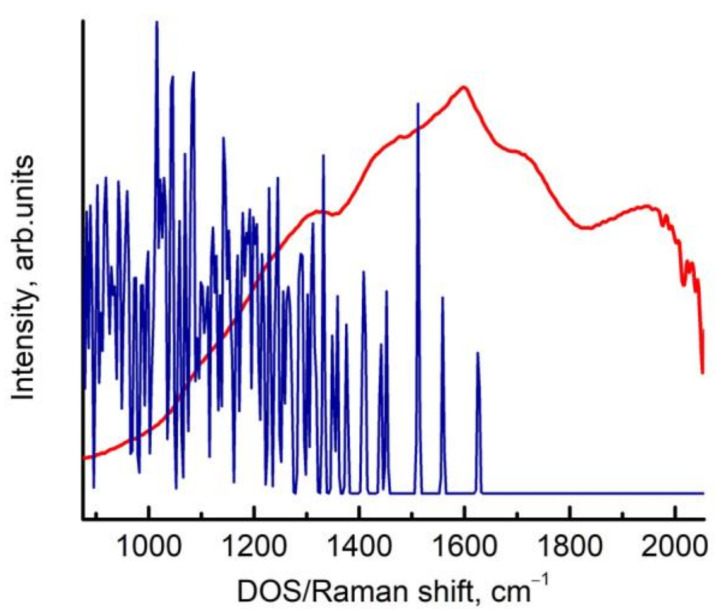
Resonant Raman spectrum and PhDOS of the 2–5 nm nanodiamonds.

Thus, all the high-frequency modes (compared to 1333 cm^−1^ for bulk diamonds) are caused by the surface Tamm states. Accordingly, the resonant effect is observed when excited at a wavelength that coincides with the band gap at approximately 1 eV of the surface Tamm states in energy. The highest frequency in the PhDOS is 1628 cm^−1^, which corresponds to the measured Raman line at 1630 cm^−1^ when excited by a 257 nm laser. Consequently, the higher modes most likely belong to the second order of the PhDOS in the 850 cm^−1^ region (Figure 2a). This is illustrated in Figure 3, where the resonant Raman spectrum and the PhDOS are given.

Unfortunately, the high-frequency part of the spectrum above 1960 cm^−1^ is hidden by the absorption of water vapor in the air, making it difficult to fully analyze the second-order resonance spectrum.

The band at 1100–1250 cm^−1^ obviously corresponds to the PhDOS (Figure 2). However, we cannot yet explain the contribution of this part of the PhDOS to the Raman spectrum of the nanodiamonds.

The distinctive feature of the obtained density of states is the presence of relatively flat high-frequency modes above 1500 cm^−1^. This behavior is similar for small and large clusters, as both have reconstructed surfaces causing such high-frequency vibrations due to the presence of carbon bounded to three neighboring atoms. The relative intensities of the low-frequency peaks with respect to high-frequency will change in favor of the former. In principle, the general shape of the phonon DOS will not change with an increasein the size of the cluster, as the high-frequency modes will still be presented. We considered a small cluster as a representative case to show the phonon DOS of reconstructed clusters.

**Figure 4:**
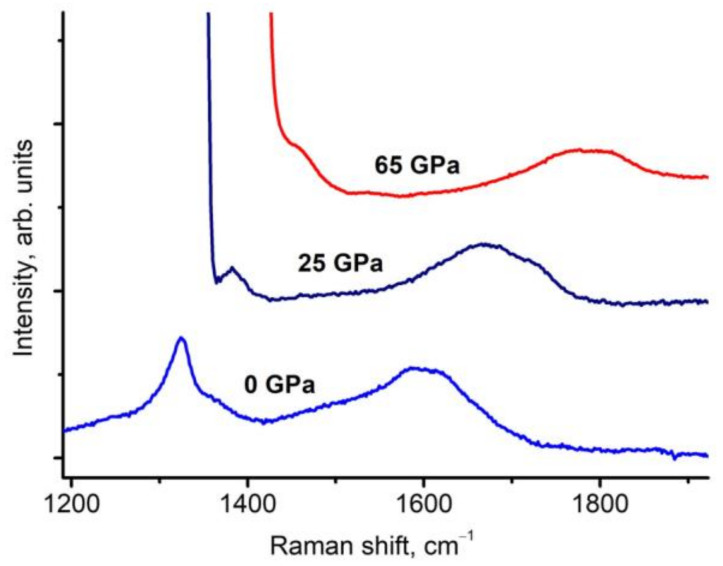
Transformation of the Raman spectra of the 2–5 nm nanodiamonds under pressure up to 65 GPa exited with 405 nm. The spectra show a part of the line from the stressed tip of the diamond anvil with a high content of ^13^C.

To verify the assignment of the 1600 cm^−1^ band to the sp^3^ bonds, we present data on the transformation of the Raman spectra of the 2–5 nm nanodiamonds under pressure up to 65 GPa (Figure 4). The Raman bands at 1325 and 1600 cm^−1^ are well traced up to 65 GPa (the highest pressure in the experiment) in the spectra. The spectra also show part of the line from the stressed tip of the diamond anvil with the high content of ^13^C, as discussed above in the Materials and Methods section. According to the dependence of the position of these lines on the pressure in Refs. [1,2], the bulk modulus of 607 GPa of the 2–5 nm nanodiamonds was obtained.

The presence of the shifted 1600 cm^−1^ band at the pressure of 65 GPa indicates that it cannot belong to sp^2^-bonded carbon. Typically, sp^2^-bonded carbon structures (graphite, glassy carbon, nanotubes, etc.) at room temperature transform to sp^3^-bonded structures at a pressure of ~15–50 GPa [40,41,42,43,44,45]. In particular, the crystalline phase of sp^2^-bonded carbon (graphite) transforms to sp^3^-bonded carbon (diamond) at a pressure of ~17 GPa [46,47]. After the graphite–diamond transformation, disordered sp^2^-bonded carbon still remains in the sample. The maximum pressure to which amorphous sp^2^-bonded carbon exists was studied in Ref. [39]. As an amorphous material, glassy carbon was chosen, the direct transition of which into diamond is not observed. Synchrotron X-ray Raman spectroscopy revealed that there is no sp^2^-bonded carbon under pressure above 40 GPa [39].

In the pressure range of sp^2^-to-sp^3^ transformations (15–50 GPa), the linear dependence of the Raman G band of sp^2^-bonded carbon on pressure breaks off. Most likely, a band corresponding to the disordered sp^3^-bonded carbon is formed in this frequency region under a pressure of ~40 GPa. The half-width of this new band is several times larger than the half-width of the G mode, and the dependence on the pressure changes dramatically. In Ref. [48], this phenomenon was called pseudomelting, and later in Refs. [39,40,41,42,43,44,45], it was shown that this effect is due to the transformation of sp^2^- to sp^3^-bonded carbon. However, for the 1600 cm^−1^ mode of the 2–5 nm nanodiamonds, the linear dependence on the pressure remains at least up to 70 GPa [1,2], and the half-width does not change noticeably with increasing pressure (Figure 4).

## 4. Conclusions

A comprehensive investigation of the Raman spectra of nanodiamonds with a size of 2–5 nm was carried out. The full Raman spectrum of the 2–5 nm nanodiamonds consists of bands located at the frequencies of 1325 and 1600 cm^−1^; also, a band at 1100–1250 cm^−1^ was observed and a plateau with a width from 1420 to 1630 cm^−1^.

The high-pressure study of the Raman spectra also shows the presence of a 1600 cm^−1^ band at a pressure of 65 GPa, which indicates that this band cannot belong to sp^2^-bonded carbon.

The resonant Raman scattering is observed at an exciting wavelength of 1064 nm. At the same time, the relative intensities of the bands at 1100–1250 cm^−1^, 1600, and 1500–1630 cm^−1^ increased fourfold, and additional lines appear expanding the previously observed dispersion interval of 1500–1630 cm^−1^ to 1423–1713 cm^−1^ (range 1630–1713 cm^−1^ refers, most likely, to the second order of phonon density of states).

The effect of the resonant Raman scattering of the 2–5 nm nanodiamonds when excited at a wavelength of 1064 nm (1.16 eV) is explained by the presence of the surface Tamm states with the band gap at approximately 1 eV.

The phonon density of states (PhDOS) for the nanodiamonds was calculated using a trained MTP. It is shown that all the high-frequency modes (compared to 1333 cm^−1^ for bulk diamonds) are caused bythe surface Tamm states.

The presence of the high-frequency modes in agreement with the lattice dynamics indicates the existence of bonds with force constants higher than in single-crystal diamonds, which could explain the possible increase in the strength of nanopolycrystalline materials.

## Data Availability

The data presented in this study are available on request from the corresponding author.
